# NIA DIVISION OF GERIATRICS AND CLINICAL GERONTOLOGY

**DOI:** 10.1093/geroni/igad104.0835

**Published:** 2023-12-21

**Authors:** Basil Eldadah

**Affiliations:** NIA, Bethesda, Maryland, United States

## Abstract

Dr. Eldadah will discuss research priorities for the NIA Division of Geriatrics and Clinical Gerontology. Dr. Eldadah will also be available for small group discussion.

